# Cutaneous lesions of bacillary angiomatosis

**DOI:** 10.1590/0037-8682-0101-2022

**Published:** 2022-08-12

**Authors:** Seow Chee Keong, Gan Wee Fu, Hasmah Hashim

**Affiliations:** 1 Hospital Melaka, Internal Medicine Department, Melaka, Malaysia.; 2 Hospital Melaka, Pathology Department, Melaka, Malaysia.

A 23-year-old man presented with a 1-month history of fever and a generalized body rash. The patient had an underlying human immunodeficiency virus (HIV) infection with a recent cluster of differentiation (CD) 4 T-cells count of 8 cells/mL and HIV-1 ribonucleic acid (RNA) 83,111 c/mL. Skin examination revealed numerous red-purplish skin papules and exophytic nodules (Figure 1A), with the largest measuring 3 cm in diameter (Figure 1B), distributed over whole-body surfaces. Hematoxylin and eosin (H&E) staining of biopsy specimens from skin lesions showed a circumscribed mass composed of proliferating capillaries with marked edema and necrosis on the surface (Figure 1C). Multiple bacilli were present throughout the mass and showed positive staining on Gram, Warthin-Starry (Figure 1D), and Giemsa. A diagnosis of bacillary angiomatosis (BA) was established following a skin biopsy. The skin lesions improved after a month of treatment.

**FIGURE 1A:**
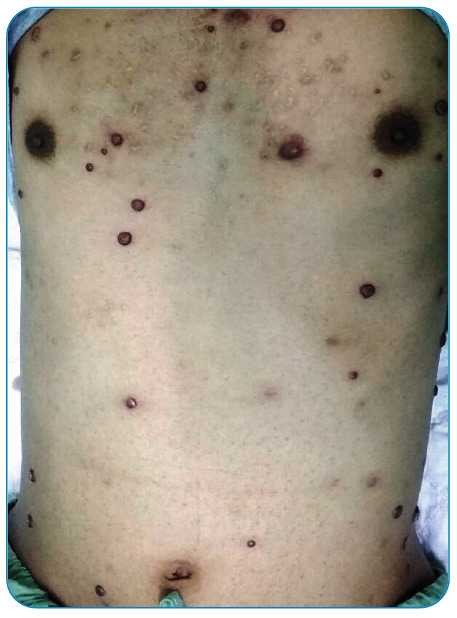
Multiple skin nodules present over the trunk.

**FIGURE 1B:**
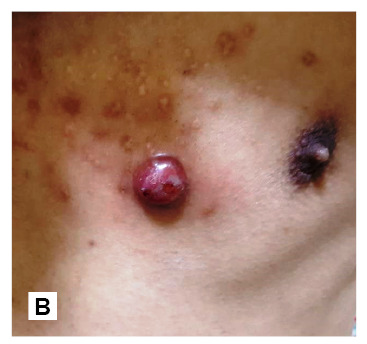
Skin biopsy is done from this nodule.

**FIGURE 1C:**
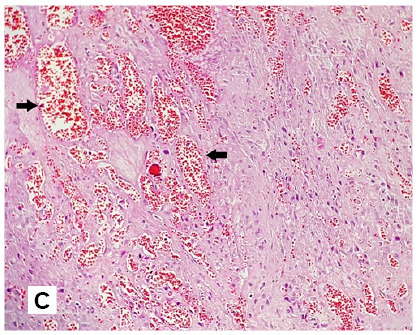
Multiple small blood vessels and ecstatic vessels filled with red blood cells (arrows) are seen under a microscope with hematoxylin and eosin (H&E) stain.

**FIGURE 1D:**
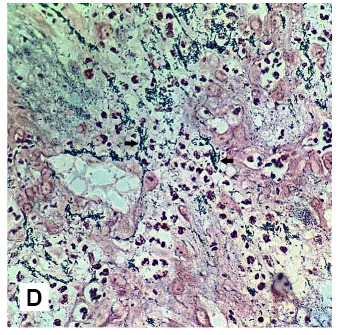
Multiple bacilli (arrows) are seen under a microscope with Warthin-Starry stain.

BA is an opportunistic infection in immunocompromised patients, such as those with HIV, who are undergoing chemotherapy or post-transplantation. It is caused by the aerobic Gram-negative bacilli *Bartonella henselae* and *B. quintana* ^1^. Skin lesions of BA can be mistaken for Kaposi’s sarcoma or pyogenic granuloma. Thus, a skin biopsy is paramount to establishing a diagnosis. Diagnosis can also be rapidly established using polymerase chain reaction assays, serologic testing, or electron microscopy. The drugs of choice for the treatment of BA are usually doxycycline or macrolides^2^. Combination therapy may be necessary for patients with severe diseases.

## References

[1] Rose SR, Koehler JE, Bennett JE, Dolin R, Blaser MJ (2020). Mandell, Douglas, and Bennett's Principles and Practice of Infectious Disease.

[2] Panel on Opportunistic Infections in Adults and Adolescents with HIV (2021). Guidelines for the Prevention and Treatment of Opportunistic Infections in HIV-Infected Adults and Adolescents: Recommendations from the Centers for Disease Control and Prevention, the National Institutes of Health, and the HIV Medicine Association of the Infectious Diseases Society of America.

